# Mapping the ethical landscape of carbon capture and storage

**DOI:** 10.1007/s10202-012-0117-2

**Published:** 2012-12-05

**Authors:** Philip Boucher, Clair Gough

**Affiliations:** Manchester, UK

## Abstract

This article describes a method of scoping for potential ethical contentions within a resource constrained research environment where actor participation and bottom–up analysis is precluded. Instead of reverting to a top–down analytical structure, a data-led process is devised. This imitates a bottom–up analytic structure in the absence of the direct participation of actors, culminating in the construction of a map of the ethical landscape; a high-resolution ethical matrix of coded interpretations of various actors’ ethical framings of the technology. Despite its limitations, which are discussed, the map can subsequently support the identification of areas where ethical contentions may be raised. Here, the method is described with reference to the construction and analysis of a map of the ethical landscape of carbon capture and storage technology. Taken as a preliminary stage of a larger study, it can support the design and initiation of more sophisticated analyses which may integrate stronger bottom–up participation and facilitate a reflective, deliberative process amongst actors.

## Abstract (de)

Dieser Beitrag beschreibt ein Verfahren, welches ethische Aspekte innerhalb solcher Forschungsvorhaben erschließt, die durch den Wunsch nach Berücksichtigung des Akteursstandpunkts bei begrenzten Ressourcen gekennzeichnet sind. Dabei wird hier eine datengeführte Bottom–up-Analyse statt eines Top–down-Ansatzes verfolgt. Sie simuliert zunächst lediglich die breite und unmittelbare Partizipation, indem sie die vielfältigen moralischen Standpunkte von Akteuren interpretiert, gruppiert und in eine komplexe ethische Matrix überführt. Trotz mancher hier diskutierter Einschränkungen lassen sich mit dieser Matrix kritische Technikbereiche identifizieren, in denen ethische Probleme zu erwarten sind. Im vorliegenden Fall wird das vorgeschlagene Verfahren am Beispiel des carbon capture and storage erläutert und angewendet. Darüber hinaus lässt sich diese noch vorläufige Methode zu einem anspruchsvolleren Verfahren ausbauen, das mehr partizipative Elemente enthält, um eine reflektive Deliberation zwischen den Akteuren zu realisieren.

## Résumé

Cet article décrit une méthode pour cadrer les contentions éthiques potentielles dans un environnement de recherche contraint par la ressource en question, et où la participation des acteurs et l’analyse « bottom–up » est écartée. Au lieu de retourner à une structure analytique « top–down », un processus mené par les données est conçu. Ceci imite une structure analytique « bottom–up » en l’absence de la participation directe des acteurs et culmine dans la création d’une carte du paysage éthique: une matrice éthique en haute résolution contenant les interprétations codées des différentes visions de la technologie des acteurs. Malgré ses limitations, qui font sujet de discussion, cette carte peut ensuite aider l’identification de lieux où des contentions peuvent survenir. Ici, la méthode est décrite en référence à la construction et l’analyse d’une carte du paysage éthique de la technologie relative au captage et stockage du dioxyde de carbone. Pris comme un stade préliminaire d’une étude plus large, la méthode peut soutenir la conception et l’initiation d’analyses plus sophistiqués qui sauront peut-être intégrer une plus forte participation « bottom–up » et faciliter un processus réflectif et délibératif parmi les acteurs.

## Introduction

Actors have varying and dynamic perspectives upon how a technology may conform with or deviate from the ethical principles they hold. Whilst many ethical principles are shared, they are not universally upheld and change over time. An actor’s perspective on how a technology relates to their ethical principles is described as an *ethical framing*. These occupy a broader *ethical landscape* of the technology which is as varied and dynamic as the sum of ethical framings of a technology. Ethical landscapes are a complex-, dynamic- and context-dependent social reality and have an important role in shaping whether and how a technology may develop. By interpreting, documenting and considering the ethical landscape of a technology, we can scope for potential ethical issues and develop greater understanding of the issues that matter to a variety of actors. Such social understandings of technology are increasingly valued in recent years. This article describes a method of mapping the ethical landscape of carbon capture and storage technology (CCS) at a particular scale by interpreting various actors’ ethical framings.

CCS is a suite of technologies which can reduce the carbon emissions associated with various processes, including coal and gas-fired electricity generation and other energy intensive industrial processes, by capturing carbon and sequestering it in secure geological formations. The UK Low Carbon Transition Plan (Department of Energy and Climate Change 2009) sets out the UK government’s strategy for reducing greenhouse gas emissions by 80 % by 2050, adopting a cumulative emissions budget. Forty percent of the UK’s electricity must come from low-carbon sources by 2020, and the electricity grid should be largely decarbonised by 2050. The specific low-carbon energy technologies that may be deployed in this task are at different stages of maturity; although there are currently no CCS demonstration plants in the United Kingdom, the technology has been identified as an important tool in reaching targets. The UK government has stated its ambition to become a world leader in CCS technology and in April 2012 announced a funding package including £1 bn capital funding through its ‘CCS Commercialisation Programme’ and a further £125 m for Research and Development including a new UK CCS Research Centre.

As a low-carbon energy technology *for* climate change mitigation, CCS engenders some familiar ethical issues regarding intergenerational equity and relationships with the environment. CCS also differs from many other forms of low-carbon energy technologies such as wind or wave energy generation in that it does not reduce the *production* of CO_2_. Instead, it offers the potential to reduce CO_2_ emissions to the atmosphere in the relatively short term whilst other demand and supply side measures are developed. As such, the ethical issues associated with CCS may differ from many other low-carbon energy technologies. For example, the climate change mitigation potential of CCS is global whilst the storage is local. Some consider CCS as a low-carbon bridge between a world currently committed to significant fossil fuel use and a decarbonised energy future whilst others are concerned that the technology could prolong industrial society’s reliance upon fossil fuels, diverting resources from the development of alternative energy production systems.

The limited attention given to ethics and CCS has been principally in the grey literature. These provide an entry point to some of the issues explored here. Spahn and Taebi (2009) explored justice and CCS, and a Corporate Watch (2008) report evaluated a variety of climate change mitigation options, including CCS, against a set of ‘ethical benchmarks’. Legget’s (2010) report on the ethics and equity issues associated with CCS framed the debate in terms of liability and legal implications, conceptualising justice in terms of acceptance, trust and social equity. The report suggested that the technology could potentially bring substantial gains to energy companies who may receive large financial subsidies from governments to develop CCS technologies whilst potentially benefiting from increased profits associated with continued demand for fossil fuels. Brown has addressed the ethics of CCS relative to storage of radioactive waste (2011) and the allocation of research funding (2008). It is important to understand how CCS is framed by various groups, as demonstrated by the successful lobby against a CCS development in Barendrecht (Brunsting et al. 2011). More recently, McLaren (2012) has explored CCS in relation to the potential procedural justice issues that might apply, considering where and why potential impacts might arise along the pathway between R&D and policy to decommissioning of storage sites. In his analysis, McLaren distinguishes localised, site related impacts and generic, typically indirect impacts (such as for example, the impacts of coal mining, or on energy markets). Furthermore, the EU Framework Programme for Research and Innovation embeds a definition of ‘responsible research and innovation’ which demands that the ethical dimensions of innovation are considered from inception and that the expectations, interests and values of all societal actors are considered.

The aim of this project is not to deliver an ethical analysis of CCS per se. Rather, it is to document and analyse the breadth of ethical attitudes and scope for areas where the technology may engender ethical contentions. A secondary aim is to devise a suitable methodology to support such an analysis under the resource constraints. The results of the analysis are considered in detail elsewhere (Gough and Boucher 2012). The focus of the present article is the methodology itself, which develops an existing ‘ethical matrix’ approach. The following section introduces the ethical matrix, and the subsequent section describes how it has been developed into a mapping approach, and how this can be used to support the identification of potential ethical issues. The final section offers concluding remarks which acknowledges the limitations of the approach and highlights the potential benefits of extending the method to a fully participative and deliberative approach.

## The ethical matrix

Our method is a development of an existing matrix approach to structuring ethical deliberations around new and emerging technologies. Introduced by Mepham (1996, 2000), ethical matrices are constructed by positioning various ethical principles on one axis and various actors on the other axis of a matrix and then populating the cells with perspectives upon the technology with respect to each actor and each ethical principle.

Studies have taken different approaches to selecting actors, defining principles, and populating the matrix. Most frequently, Beauchamp and Childress’ (2001) four ‘classic principles’ of justice, autonomy, beneficence and non-maleficence are used, although the latter two are often collapsed into a single principle of well-being. Cotton (2009a, b) highlighted that the approach adopts a top–down analytical structure where the lists of actors and principles, which define the boundaries of the analysis, are selected by the researcher. He suggested moving towards a bottom–up analytical structure by increasing the level of participation of actors. Actors would not only complete the cells but also define the lists of actors and principles that structure the matrix and define the boundaries of the study. Such an approach would improve the legitimacy of analysis and afford the participants greater ownership of the matrix, supporting a deliberative process. For it to work, however, the lists defining the boundaries must be broadly comprehensible, clear, simple, consensual and limited in number. This is particularly important in the list of ethical principles, which draws upon sophisticated concepts articulated in terminology which has developed over centuries of esoteric debate. This complexity may restrict proper participation and hamper the deliberative process.

The matrix approach remains somewhat embryonic but the concept has been examined and applied in a growing range of case studies. Empirically, these are dominated by topics pertaining to food (e.g. Mepham 2010; Kaiser et al. 2007) and environmental issues (Gamborg 2002; e.g. Cotton 2009b; Oughton et al. 2004). The ultimate aims of matrix analyses also vary across studies. Some are designed to guide deliberation and improve the penetration of rational ethical analysis into decision-making processes (Mepham 2000) whilst others, particularly those with a bottom–up analytic structure, can also be seen as an end in themselves, facilitating deliberative engagement amongst actors (Cotton 2009a, b). The results of ethical matrix analyses have generally been positive, with encouraging reports from various public and elite participants.

Perhaps, the case which exhibits the most similarity with the present study is Cotton’s (2009b) consideration of the suitability of the ethical matrix approach in the context of the siting of a radioactive waste management (RWM) facility. Whilst CO_2_ does not pose the same risks as radioactive material, both CCS and RWM involve storing by-products of less carbon intensive energy production techniques. Clearly, RWM and CCS are associated with very different energy production technologies, and there are many differences in how actors frame these technologies. Yet, both combine widespread, finite and benefits of low-carbon energy production with localised consequences in the maintenance of the storage site. As such, some similar ethical issues, such as intergenerational equity and environmental justice, may be raised.

## Mapping the ethical landscape of CCS

Certain structural features of the current project have shaped its specific approach to the ethical matrix. The first is that it is not supported by significant time and resources. This limitation precludes the establishment of a meaningful engagement with the actors. As such, the research is *not participative* and relies heavily upon secondary data. This certainly places limitations upon the legitimacy and robustness of the results. However, a desk-based approach also presents some opportunities. Principally, it allows us to explore the potential for a data-led analytical structure, integrating some of the advantages associated with a bottom–up approach via an essentially top–down mechanism, pushing the boundaries of what can be achieved with limited resources. It also allows the research team to indulge in a matrix of greater complexity and size without concerns about its comprehensibility to participants. The second structural feature is the aim to develop understanding of CCS’ ethical landscape and scope for areas of potential ethical contention. It is not intended to facilitate deliberative engagement amongst actors, but it could provide a step towards greater understanding amongst technical elites. It is not anticipated to support a decision-making process, but we anticipate further studies that should be able to inform development. We designed the method so that it can be used as a preliminary study leading directly to the design and initiation of an analysis with grander aims. These limitations, opportunities and potential further improvements are all considered in greater detail.

The relevant actors and principles are each in flux, subject to change as new actors enter the debate and existing actors change their positions, leave the debate and form implicit or explicit coalitions. Any method of mapping must be sufficiently flexible to cope with the dynamic character of the ethical landscape, particularly with ‘hot’ or controversial technologies which may have a more rapidly changing ethical landscape. To meet our aim of scoping out the ethical landscape, we must iteratively develop the matrix until a satisfactory ‘snapshot’ is produced at an appropriate scale. The remainder of this section describes the method that was developed towards our particular aims and within our particular resource constraints.

### Mapping ethical landscapes

Now, recall that the rows of ethical matrices capture ethical perspectives with *respect to* each actor. This means that any actor can be identified, including those that cannot or do not hold or articulate a framing of the technology themselves such as future generations or non-human actors. The ethical issues are considered for them via *someone*’*s* understanding of their interests; often, the researcher or some other actor participating in the research. Here, we are not trying to document all ethical features for each actor but to understand what the actors’ own ethical framings of the technology are, and identify areas where ethical contentions may be raised. This is a subtle but important difference, and it leads to a different approach to populating the matrix. We use the rows to capture each actor’s *ethical framing* of the technology directly; how *they* position the technology in compliance with or deviation from the ethical principles. Whilst these framings may be held through empathy or respect for another actor, they are recorded as their own framing and not those of the other. This approach removes an interpretive layer between the provenance of the ethical framing and its positioning in the matrix, improving the traceability and legitimacy of the analysis. This is particularly important in non-participative studies. The approach has some consequences for the treatment of non-human actors in our study, which will be revisited.

Ethical analyses draw upon three broad traditions. The first is *utilitarianism*, which advises us to take those actions that provide the greatest utility for the greatest number. So, benefits or hedonistic pleasures are balanced against net costs. In the contemporary literature, these teleological approaches are more often associated with social contracts and community outcomes. A second *deontological* approach contrasts with utilitarianism’s focus on outcomes by basing ethical judgements upon procedures and processes. A third tradition, *virtue ethics*, accounts for different interpretations of, for example, what the benefits, costs and correct procedures *are*, focusing upon the qualities and characteristics of actors. To illustrate with reference to CCS, a utilitarian perspective may highlight the ethical acceptability of CCS by outweighing the local storage costs against the global benefit of climate change mitigation. A deontological approach may focus upon the legitimacy of the development process or the propriety of technical fixes for climate change. A virtue ethics approach would consider the ethics of the technology with reference to the relevant actors and their contexts, histories and relationships. Here, we do not aim to produce an ethical judgement per se but to gather other actors’ ethical framings. These framings may be implicitly aligned with any of these traditions and might not be applied by the actors in a consistent way. In producing the map, the researchers must be aware of these different ways of thinking about ethics and sensitive to different foci of ethical perspectives.

The participative approach espoused by Cotton (2009a, b) allows the actors to define the principles that structure the analysis. Such a bottom–up or actor-led approach is clearly not possible here where only secondary data is used. Rather than revert to a top–down approach where the research team select the actors and principles, we developed an approach where the boundaries of analysis are defined through a process that is led by the interpretation of material produced by the actors. As such, this data-led approach imitates bottom–up analytical structure via a top–down mechanism.

This approach also overcomes some of the constraints associated with the bottom–up approach. Since the matrix is no longer restricted by the constraint of being sufficiently small and comprehensible to a wide variety of actors, it can accommodate whichever ethical principles emerge as important, including unusual, disputed, emergent, esoteric or otherwise ‘fringe’ ethical principles. This allows a more sophisticated understanding of the issues that are raised. The actor axis of the matrix can also be extended significantly to include *any* actor articulating a position which can be interpreted through the lens of *any* ethical principle. Unrestricted by the complexity associated with wide inclusion, extending the matrix beyond the 3 × 3 or 4 × 4 format, we produce a high-resolution matrix which can then be coded to produce a broad, visual map of the ethical landscape. This can then be traversed to meet the ultimate aim of identifying areas where ethical contentions may be raised.

We argue that our desk-based, data-led approach can achieve a great deal with limited resources. Whilst it is no substitute for a fully participative study, it does provide a ‘first look’ at the ethical issues associated with a technology, either to flag potential issues or to support the design of more detailed follow-up analyses. The methodological process can be described in a series of steps organised into three key phases. The first phase is preparatory, identifying an initial set of principles and actors to define the initial boundaries of the map and to provide an entry point to the data. The second phase is the data-led construction of the map, adjusting the list of principles and actors to reflect the ethical landscape and populating the map with coded descriptions. This step is repeated iteratively until the map is considered to adequately capture a representative range of perspectives. The final phase is analytical, considering pertinent features of the map to identify and explore potential ethical issues. A mindful, reflective attitude should be adopted throughout and the whole process should be well documented. The following three subsections describe each phase of this methodology in turn, illustrated with reference to our analysis of the ethical landscape of CCS.

### Phase 1: setting initial boundaries

Before the construction of the map, an initial set of principles and actors is required to initiate an opening dialogue with the data. This set will then be adjusted in a data-led process. The selection at this stage is of a top–down character, and the research team must document the process and provide justifications for all selections. The aim is to provide sufficient breadth from the outset to allow maximum inclusivity without constraining or overdetermining the issues ex-ante. Since we are not limited by size, exploration and experimentation are encouraged in anticipation that this will lead to greater inclusivity of ethical framings. Once the initial lists have been selected, significant change is expected with many being removed, added and redefined in the data-led process.

#### Identify initial set of ethical principles

The first step is to identify an initial set of ethical principles which actors are likely to hold as relevant to the technology. This list forms the starting point from which the final set of principles evolves. Many studies are limited to Beauchamp and Childress’ (2001) ‘classic’ principles of justice well-being and autonomy. These, however, were designed in a bioethical context. Here, since we are not limited by size or complexity and aim for a high-resolution representation of the ethical landscape, we adjust these to suit our context and introduce many other principles too. The key resources at this stage were the literature on applied ethics, particularly in a technical context (e.g. Wilcox and Theodore 1998; Palm and Hansson 2006); the ethical matrix literature discussed above (e.g. Mepham 1996); ethical analyses pertaining specifically to CCS (e.g. Legget 2010); and the research team’s existing familiarity with some stakeholders’ perspectives on the technology, gained through previous research. The principles and their origins were discussed amongst the researchers to ensure a common understanding of their meaning. Note that the actors might not hold the understanding of these principles as defined in the literature. It is more important to allow the matrix to capture the actors’ ethical framings as accurately as possible. The initial list is presented below with a brief discussion of their selection. These principles and their definitions are all subject to change in the subsequent data-led process.

Four principles of justice: Justice is one of the four ‘classic’ principles (Beauchamp and Childress 2001) and has been used directly in many previous matrix analyses (since Mepham 1996), although it is occasionally listed as *fairness*. We felt that there may be a number of different dimensions of justice which could be worth differentiating. Four such dimensions often associated with environmental and infrastructural issues are *intergenerational*, *social*, *environmental* and *financial*. We included all four as separate ethical principles, grouped together within a broad theme of justice.
Two principles of well-being: Cotton (2009b) took two of Beaucham and Childress’ (2001) classic principles; providing benefits or *beneficence* and preventing harm or *non*-*maleficence*. He then combined them in a single principle of well-being. We include these principles individually, but couple them in a theme of well-being.
Autonomy: Beauchamp and Childress’ (2001) fourth classic ethical principle is also included directly in our selection.
Honesty: This principle, described in the applied ethics literature (Wilcox and Theodore 1998), was added to capture ethical issues around the transparency of communications between different actors, for example, stakeholders, specific communities and wider publics.
Trust: We added this principle to capture whether actors felt CCS complied with or deviated from a value of trustworthiness.
Naturalness: This principle captures whether actors felt CCS complied with or deviated from actors’ understandings of nature and how society relates to it.
Competence: A principle of competence was found in the applied ethics literature (Wilcox and Theodore 1998), capturing whether technical and managerial practices meet expected standards to ensure the effective and safe operation of developments.
Social values: This principle, adapted from Palm and Hansson (2006), captures how the technology relates to social values such as the ways in which people understand themselves, other people, technologies, practices, biota, environments and others. It also captures how the technology may engender changes to these values. Of course, social values are heterogeneous and any actor articulating that the technology deviates from or transforms *any* group’s social values would be recorded as part of this actors’ ethical framing of the technology.

#### Identify initial set of actors

This step, like the first, seeks to provide a starting point for the process which will come to define the boundary of the map. Because of our existing knowledge of UK stakeholders and the limited resources available for the study, we decided to focus upon UK actors, although a number of the actors operate at an international scale. We were familiar with some actors through exposure to a range of stakeholders gained during previous research. To capture as much of the breadth of ethical perspectives as possible, we selected actors from across organisational spectra, ensuring representation from NGO, governance, industrial and public communities. These categories are not used to provide an a priori structure for the list of actors or the analysis.

There is no reason why the interests of non-human actors such as fauna, future generations or natural properties such as biodiversity cannot be represented in the map, although there are some points to consider arising from our actor list. As discussed, actor lists on ethical matrices (e.g. Mepham 2000) usually capture ethical issues with *respect to* a number of actors. This differs from our approach, where each row captures an actor’s ethical framing of the technology directly. This affects how ethical concerns for other actors, such as non-humans and future generations, are captured. We need not speculate whether these actors hold ethical framings. The point is that they are not articulated in a format which we can interpret. Their direct inclusion of is precluded because our matrix is a compendium of ethical framings ascribed directly to those that articulate them, so they cannot breach our selection method. This is by design for two key reasons. First, it removes an interpretive layer between the provenance of an ethical perspective and its representation in the map. Current generations’ concerns for future generations are ascribed to those that hold them, not those that cannot. This gives a more accurate description of the actors’ framings, befitting our aim of understanding and representing ethical perspectives. Second, it suits our aim of identifying areas where ethical contentions may be raised by actors. A classic ethical analysis aiming to identify potential ethical dilemmas regardless of their manifestation in wider debates should adopt Mepham’s top–down approach.

Some actors’ ethical framings are borne out of respect for other actors, including future generations and biodiversity. As such, we include actors who may position themselves as representatives of the interests of other actors, such as Christian Aid on future generations or WWF on biodiversity. This means that the Christian Aid perspective on CCS and intergenerational justice is recorded as just that and is not extended as a voice for future generations themselves. Likewise, a WWF perspective on environmental justice may be articulated as a proxy for the interests of endangered species, but we record it as part the WWF ethical framing. In doing so, we transfer the interpretive process of respecting the interests of other actors from the research team to the actors in the study. Note that, some of our initial set of principles is also designed to capture values; we think might be relevant to these actors, for example, intergenerational and environmental justice. This is to allow the greatest opportunity for all articulated ethical concerns to be captured in the map.

Care must be taken when collapsing heterogeneous groups such as ‘local communities’ into single actors which may have a diverse or even discrete range of concerns. Indeed, all organisations are expected to have internally varied perspectives upon a technology’s relationship with ethical principles, even if this is not reflected in their public facing organisational perspective. Without multiple access points to each actor, it will be difficult to capture this heterogeneity.

Ethical landscapes can be analysed at different scales. Mapping an international ethical landscape would require a global network of localised researchers, communicating regularly with each other to understand the relationship between different actors’ understandings of principles and the technology’s compliance with and deviation from them with sensitivity to the significant cultural and linguistic diversity that would be encountered. This may lead to more gaps, reflecting the greater variety of principles upheld over larger scales. Smaller scales could also be considered, such as facility siting controversies. This may be an opportunity for deeper analysis of the internal dynamics of actors—how an organisational position is reached—which may be intractable on a larger scale. This would require multiple points of access to each actor with significant participation. Clearly, not *all* individuals’ ethical framings can be practicably captured in a single matrix. Such detail would be intractable for larger national or international ethical matrices. The selection of initial actors should be balanced and targeted to meet the empirical aims of the research. Whilst we endeavoured to take into account the international nature of CCS development, and geographical variations in the ethical analysis, we adopted a national scale focusing UK framings of the technology. Each self-identifying actor is represented with a single row of the matrix. Broader analysis would necessitate wider cultural and linguistic resources in the research team. Deeper analysis would require significant participation with multiple points of access to each actor.

### Phase 2: iterative development

This is the central phase of the analytical process, in which the map is populated with the actors’ ethical framings, and the boundaries of analysis are adjusted in a data-led process.

The phase is iterative because changes to the boundaries of the map, defined by the lists of actors and principles, will lead to changes in how the map should be populated. This continues until a satisfactory map, a snapshot of the ethical landscape at a scale and resolution that befits the project aims, is produced.

#### Populating the matrix

Using the most up to date material that is publicly available—largely reports, websites and press releases—the research team identify statements that can be interpreted as ethical framings. These are the statements that are used to populate the map. A qualitative reflection of the actors’ understandings of CCS in the context of each principle is entered into a cell on the matrix, referencing the empirical material which supported the analysis. Sometimes these perspectives are articulated implicitly, sometimes explicitly, but they are always justified interpretations, illustrated by referenced empirical material. If no framing is found, the cell is left blank. An explicitly neutral framing can also be recorded. It must be noted that the actors’ reasoning for the relevance of a principle or CCS’ relationship with it need not be recognised by the research team or any other actor. The point is to capture the actors’ perspectives on how CCS complies with or deviates from ethical principles that matter to them. Validity is not granted to principles or framings on the basis of their compliance with any privileged perspective—whether it is of a scientific, political, religious or other character—and no screening can occur on this basis. One consequence of this approach is that fringe perspectives may be represented as prominently as those that reflect the perspectives of the many or the powerful. This issue is revisited later.

Cells are coded to reflect the research team’s interpretation of the extent to which the actors position CCS in compliance with or deviation from each ethical principle. Other matrices have coded findings for ease of interpretation using various symbols. For example, Mepham (2000) used □ and • and Forsberg (2007) used + and − to indicate framings of *respectfor the principle* (cf. compliance) and *infringement* (cf. deviation), respectively. In order to capture neutrality and both moderate and extreme perceptions of CCS’s conformity or deviance with the ethical principle, we use 5 codes, as presented in Table 1. In our case, the angle brackets and hash keys are instructions for an automated colouring function in the spreadsheet which hosts the matrix. The darkness of the hue reflects how strongly the framing is articulated. It must be noted that, without direct participation of actors, this may also reflect the extent to which concerns are expressed directly or explicitly.

**Table 1:** Coding ethical framings of CCS

≫	The technology conforms strongly/explicitly with the ethical principle
>	The technology conforms moderately/implicitly with the ethical principle
#	The technology neither conforms with nor deviates from the ethical principle
	No statement is available in relation to the principle
<	The technology deviates moderately/implicitly from the ethical principle;
≪	The technology deviates strongly/explicitly from the ethical principle

Populating the matrix is not always straightforward. For example, the principles of providing benefits whilst minimising harm are separate, but can be considered to be ‘two sides of the well-being coin’. Whilst each is fairly straightforward, the process of balancing them against each other can be trickier, engendering questions of risk and equity. Principles such as these which capture strongly related concerns can make it difficult to distribute interpretations of actors’ ethical framings. Some speak to multiple principles and may be entered into the matrix many times in a single row. This kind of overlap is expected and is not considered problematic here as the map will not be used to provide aggregated ethical ‘scores’.

#### Adjusting set of actors

The suitability and validity of the list of actors should be constantly re-evaluated. They are adjusted in a data-led process. Actors who do not express an ethical framing of the technology, implicitly or explicitly, are removed from the actor list. Additional actors are identified through a ‘snowballing method’ of searching through data, and adding actors who are mentioned. Where actors refer to other actors in their material, these are investigated and considered for inclusion in the matrix. Their inclusion will depend upon the availability of resources for analysis and the content of such material; whether it expresses an ethical framing and whether inclusion is commensurate with the aims, scale and boundaries of the study.

#### Adjusting set of principles

The initial list of principles was produced from the top–down, reflecting insights from the literature combined with the researchers’ ex-ante judgements of the landscape. The list should be adjusted in a data-led process. In doing so, the researchers must continually reflect upon and document their role in the process. Maintaining an appropriate balance of principles is a delicate process. Principles for which no response can be found are removed. Where ethical concerns are raised in the material that do not appear to fit any of the defined principles, the research team should revisit the literature and adjust the list of principles to capture ethical framings as faithfully as possible. The definition of principles which do not adequately capture the actors’ framings should be reconsidered and possibly adjusted. Where a principle is drawn upon in disparate fashion, capturing different types of ethical framing, the principle should be split into two or more separate principles. Similarly, two or more principles which capture the same aspect of actors’ ethical framings can be merged into a common principle. Any of these adjustments may have knock-on effects upon other areas of the matrix. The remainder of this section describes how the initial list of principles was adjusted in our research, culminating in a discussion of those that came to define the boundaries of the completed map.

We identified the need for a principle which could capture concerns about who would hold long-term responsibility for the technology and its impacts. We decided that a principle of accountability should be added to the matrix to capture this dimension of the ethical landscape. Similarly, a principle of propriety was added to capture actors’ understanding of the ‘rightness’ of the technology. This principle was thematically linked to the existing principle of naturalness. The principle of social values was removed, as all relevant responses to it were captured by the more specific principles of propriety and naturalness. We grouped these principles in a theme; *human understanding and social values*. The principle of trust was removed because the ethical perspectives it captured were duplicated in other more specific principles, most notably competence, honesty and the newly added principle of accountability.

Initially, a single principle of competence was used to capture whether scientific, technical and managerial practices and knowledge were of a sufficient standard to ensure the effective, safe and reliable operation of developments. When considering actors’ responses to this principle, we identified a distinction between technical and social facets of competence. It was felt that the difference was important and having a single principle to capture all of these perspectives restricted the capacity of the matrix to construct an appropriate map of the ethical landscape. The principle was split into two new principles of managerial/regulatory competence and technical/scientific competence which were grouped together within a theme of competence. The introduction of this additional distinction within the principles led to some knock-on readjustments of framings, which the authors were confident, delivered an adequate map of the ethical landscape in a format suited to the analysis required.

In this final iteration of the matrix, the principle of naturalness captures a relatively small feature of the ethical landscape. The research team considered whether the principle of naturalness should remain, deciding that it should. The principles would be removed if Bellona, the only actor in our list engaging this principle, adjusted their perspective or aligned it more closely to another principle such as propriety. On the other hand, if further analysis revealed more actors engage the principle, perhaps based upon slightly different understandings of naturalness, for example, geological nature, biotic nature, social nature or spiritual nature, then it might eventually need to be expanded to allow it to capture ethical framings in appropriate detail. The analyst should not pre-empt such revisions, but allow the data to lead the process.

The thirteen principles were organised into four themes which developed, principally, through the division of relatively wide principles such as justice into more specific sub-principles. These themes are not used to structure the analysis, but are included to provide a richer presentation of how the researchers understand each of the principles. A common understanding of the meaning of each principle was maintained through regular discussions amongst the research team. Definitions were written in the form of questions that each principle asks of the technology via the data.

##### *Principles of justice*

Intergenerational justice: Does CCS conform with the suspected interests of future generations and is it of greater benefit to less advantaged generations?
Social justice: Does CCS conform with the interests of all social groups and is it of greater benefit to less advantaged social groups? (The application of this principle on the matrix incorporates notions of international, developmental and economic justice.).
Environmental justice: Does CCS conform with the suspected interests of non-human species, valued environmental qualities such as biodiversity and ecological sustainability? Does the technology conform with the provision of appropriate environmental services for all?
Financial justice: Does CCS conform with an appropriate distribution of rewards, incentives and liabilities (including the financial opportunity cost investing in other technologies; demand reduction and other production options)?

##### *Principles of well-being*

Providing benefits (beneficence): Does CCS provide some benefits to any actors?
Preventing harm (non-maleficence): Does CCS prevent harm to any actors?

##### *Principles of control, influence and power*

Autonomy: Does CCS affect any actors’ capacity for self-determination and freedom to shape their own understandings and decisions?
Honesty: Is information disseminated about CCS accurate, thorough and sufficient and does it come from appropriate and balanced sources, communicated with sufficient transparency?
Accountability: Does CCS conform with the actors being responsible and accountable for the consequences of the risks they take?
Technical and scientific competence: Are scientific, technical and engineering practices and knowledge of a sufficient standard to ensure the effective, safe and reliable operation of CCS developments?
Managerial and regulatory competence: Are managerial, regulatory and legal practices and knowledge of a sufficient standard to ensure the effective, safe and reliable operation of CCS developments?

##### *Principles of social understandings and human values*

Propriety: Does CCS deviate from or transform any social understandings or human values regarding what is right and what is the right way to progress, deal with problems and search for solutions?
Naturalness: Does CCS deviate from or transform any social understandings or human values regarding nature, natural processes or human relationships with nature?

**Fig. 1:**
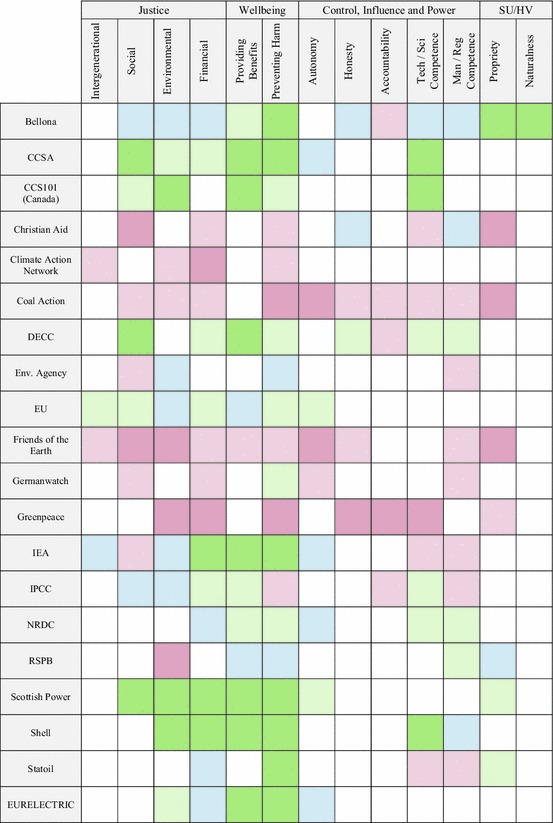
*Map* of the ethical landscape of CCS

The list should not be assumed to be exhaustive, final or adequate in other analytical or empirical contexts. This is an epistemological point because of the interpretive nature of cartography in general and the elevated interpretive role of the researcher in the absence of actor participation. It is also an ontological point because of the dynamic character of ethical landscape itself. The analyst must remain open to the need for further revisions as the landscape develops and new ethical framings emerge. The development of the map could be prolonged indefinitely to trace the development of an ethical landscape over time, with actors and principles being added and removed to reflect ongoing changes in the ethical landscape. In our case, the map was developed until we felt that it reflected the landscape at a national scale and organisational level sufficiently well to support a process of scoping for the loci of potential ethical contentions. Figure 1, below, presents the map which resulted from our analysis. A fully populated version of the matrix which contains details of the statements in addition to their allocated coding is available in an electronic appendix as an Excel spreadsheet.

### Phase 3: analysing the map

Looking across the two dimensions of the matrix, the analyst may consider each actor’s perspective upon the technology’s relationship with various ethical principles (rows) or the various actors’ perspectives upon the technology’s relationship with a specific ethical principle (columns). Cells cannot be aggregated to produce an ethical ‘result’ for the technology, or even its performance against a single principle. This is because the matrix does not capture the representativeness of a framing or how actors may prioritise conflicting principles in a given context. Similarly, we cannot take a dominant appearance of redness as an indicator of potential ethical contention because it actually represents consensus on how the technology relates to a principle. Furthermore, they may also be a consensus that this principle is overridden by other benefits or is not a priority. To really understand the importance of its features, the researcher must use the map as heuristic devise, supporting deeper, more systematic consideration of the broader ethical landscape. Each type of analyses is described in following subsections before a final subsection describes how potential ethical issues are identified. In each case, given the methodological remit of this article, we focus on how the analysis is performed rather than results and their implications for the technology.

#### Actor-by-actor analysis

Each row of the matrix describes an actor’s ethical frame of the technology; coded interpretations of how the technology conforms with or deviates from each actors’ ethical principles. These frames reflect the breadth of ethical perspectives articulated in the material. It may be interesting to consider actors who share similar ethical framings. These are described as *ethical coalitions,* and they could be important if ethical contentions are raised. The actors may not identify or even recognise themselves as an ethical coalition. They may have different reasons for their ethical framings and diverge in their broader understandings of the technology. The importance of a coalition is difficult to judge without further research to establish the robustness of the group, and its power to influence development. Two potential ethical coalitions were identified in the present analysis. The first comprised CCS101, CCSA, Scottish Power and Shell and the second Coal Action, Friends of the Earth and Greenpeace. Whilst these particular coalitions might not be surprising to those familiar with perspectives on CCS, unexpected coalitions could be identified through actor-by-actor analysis. Other ex-ante approaches to grouping actors—for example, by their sector, size or technical frame—are avoided on the basis that such categories are outside the boundary of analysis and could actually obscure features of the ethical landscape.

Analysis must be sensitive to the fact that entries are not weighted in any way. This means that framings are presented without any reference to the number of actors or individuals who hold the ethical framing or, perhaps more importantly, how much power these actors may have. Actors have varying degrees of power to shape the development of a technology, to enrol other actors, to define the boundaries of debate. The concerns of less powerful actors may need to be translated before they can be articulated in the language or discursive space in which a technology is debated and may, as a result, be under-represented (Boucher 2012). These possibilities cannot be captured in the map. The reverse concern also applies that framings representing few actors or fringe perspectives are listed as prominently as those who represent many actors or mainstream framings.

#### Principle-by-principle analysis

Each column of the matrix documents actors’ responses to how the technology is considered in relation to the given principle. Low colour variance indicates that a degree of consensus has been achieved as to how actors and coalitions associate the specific principle with the technology, that is, consensual framing of compliance, deviation or neutrality. It may be worth investigating how this consensus has been achieved, regardless of whether it is of conformity or deviation. In our study, there was consensus around CCS’ deviation from the principle of accountability and only one actor framed the technology in deviation of a principle of providing benefits, two principles that many actors upheld.

Where single columns of the matrix exhibit a high level of colour variance, it is implied that actors’ disagree on how CCS relates to the ethical principle in question. Such areas of the map are described as potential *ethical faultlines*. Faultlines may appear for various reasons. They could represent disparity, contention, openness, negotiation or flexibility amongst actors’ ethical framings. Deeper analysis is needed to consider whether such faultlines represent different understandings of the principle, the technology or how they are related. Most importantly, we suggest that they may highlight loci for the emergence of ethical issues, regardless of whether these issues are currently manifested or not. Potential ethical faultlines were identified for all four principles of justice, both principles of competence and preventing harm. These were analysed more deeply to consider the character of the faultline and whether ethical contentions may be engendered.

In addition to this isolated analysis of principles, it may also be worth comparing colour profiles across principles. Similar responses to a number of principles would indicate that actors associate the technology with these principles in similar ways. The map indicates that these principles may be linked in some way, but a fuller understanding, again, requires deeper analysis of the wider landscape. In the present study, a broad linkage may hold amongst principles of intergenerational justice, autonomy, honesty and managerial/regulatory competence and also financial justice and propriety.

#### Developing a list of potential ethical contentions

This final step is to use the analysis of the map to identify features of the ethical landscape that may lead to ethical contentions. This article focuses upon the methodology, so we present examples of the kind of analysis that is undertaken.

Areas of potential ethical contention could be identified by principles which many actors uphold and feel the technology deviates from. As discussed, the most notably deviated principle in our study is accountability. The nature of this consensus is unclear, and the actors may have different reasons for their framings. For example, this deviation may reflect the immaturity of the technology and, perhaps, issues with its regulation rather than a fundamental ethical issue with the technology per se. The implications are also unclear as actors may, for example, unite to shape a conforming development path. On the other hand, the consensus may attract less discussion and debate, reducing the salience of the issue. For these reasons, we undertook a deeper analysis of the landscape. We found that the main point of deviation is with legal and regulatory accountability for the technology. For example, that few countries have established the regulatory or legal frameworks required for long-term storage and significant regulatory issues must be addressed before the United Kingdom would be in a position to store CO_2_ from other nations. Some actors considered that the distribution of responsibilities and liabilities associated with stored CO_2_ in the longer term remains unclear.

Other areas of interest are ethical faultlines, where the actors do not agree whether the technology complies or deviates from a given principle. These are identified on the map as columns featuring high colour variance, but deeper analysis is required to understand whether the actors really position the technology in conflicting relationships with the principle. As discussed, seven of our thirteen principles are identified as faultlines; all four principles of justice, both principles of competence and the principle of preventing harm. Again, full analyses are required to understand the nature of these faultlines. For example, examining the faultline around scientific/technical competence reveals that actors are divided over whether they can trust the technology to deliver on its promises and whether the technical knowhow to ensure the long-term success of the technology can be guaranteed, this appears to be a genuine faultline in the ethical landscape. The faultline around managerial/regulatory competence reveals a difference in the area actors are focusing on; CCS developments in some nations would comply with the principle but those in others would not. As such, this might not be a genuine faultline in the ethical landscape, but the tension could still lead to ethical contention. In our final report, we concluded that there are four key faultlines in preventing harm, environmental justice and both principles of competence, as well as significant concern about accountability. Validation of these features of the ethical landscape should be sought, and the issues should be explored in greater empirical and theoretical detail.

## Discussion and conclusions

This article has described a methodology for identifying potential ethical issues by organising a large qualitative data set into a visual map of the ethical landscape of a technology. Since resource constraints precluded direct actor participation, the map is constructed in a data-led process designed to imitate a bottom–up approach via a top–down mechanism. We took advantage of the freedom of a desk-based study, particularly the liberation from a small, simple matrix to a high-resolution map which can incorporate whichever ethical principles are considered relevant. It is important not to overestimate what kinds of analysis the map can support. Whilst it is a useful heuristic device for analysis and is also a convenient format for discussion and presentation, it remains a compilation of qualitative interpretations of a sophisticated set of positions that are articulated over numerous long sources. The deeper content and context of the cells must be kept in mind when considering the ethical landscape. Further, the map produced does not represent a comprehensive catalogue of all ethical framings of CCS technology but, rather, a visual snapshot of the ethical landscape at a given scale and resolution. We suggest that the methodology is appropriate for the limited scoping aims of the current project. In this concluding discussion, we consider other uses of the methodology, including its repositioning as a pilot study for a fully participative and deliberative analysis which would provide a sufficiently thorough, robust and legitimate analysis for a project with grander aims.

Earlier, we stated that participative approaches are constrained to simple comprehensible boundaries. Liberated from this constraint, we extended the matrix significantly. We also adjusted its structure, so the content does not describe how the technology relates to a principle *withrespect to* each actor (from the perspective of another) but, instead, describes an ethical framing *from the perspective of* each actor directly. We suggest that the map produced through the methodology described here can be redefined as a pilot study and used as a the first step in a participative, bottom–up analysis which would overcome a number of limitations, improve the legitimacy and robustness of analysis and allow more ambitious aims. This would require significant investment of resources and also significant commitment from each participant.

The first advantage of participation is legitimacy. The data that led the construction of the map was drawn from documents produced for various purposes. Much of this data were not intended to be taken as a contribution to an ethical debate. In treating them as such, they may be inaccurate, incomplete or out of date. Actors can be asked to adjust, augment and most importantly validate the ethical framings presented in the pilot map. The content and coding of the cells could be completely redefined through in-depth interviews with the actors, with the pilot map used to identify important features and support discussion. Ideally, full participation would involve the actors also defining the boundaries of analysis, engaging with the list and definition of principles and suggesting more actors. This would replace the data-led pilot with a bottom–up analysis.

Currently, entries are not ranked in any way. This could be important because actors hold multiple, possible conflicting, ethical principles simultaneously. As a result, they may draw upon sets of rules to decide which principles dominate others. The priorities denoted by these rules will be as normative and heterogeneous as the ethical perspectives themselves. They are likely to be held tacitly and deployed in a context sensitive or ad hoc manner. Through participation, the actors could comment on their priorities. This could be recorded as supplementary information, alongside the map, which would support analysis. It may be possible to incorporate this to a coding in the map itself, perhaps with the actors expressing which principles matter to them, which they hold as relevant, their priorities and also the extent of compliance and deviation.

The prevalence of gaps, where no ethical framing is identified with regards a particular principle, is notable in our map in Fig. 1 when compared with the example matrix. This is because a top–down analytical structure allows the researcher to consider the relevance of any principle to any actor, but this is not the case in a data-led analytical structure, where the researcher can only interpret framings articulated by the actors themselves. Actors might not uphold ethical principles that others do or might not find it relevant to the specific technology. This kind of gap may be more prevalent at wider scales, incorporating actors who draw upon more disparate ethical frameworks, hold different understandings and values of the self, nature and other actors. As such, gaps are seen as important parts of the map and their prevalence is not a measure of failing or incompleteness, and they are an important part of the representation of the ethical landscape. Other forms of gap, however, may feature where actors hold an ethical framing but do not articulate it explicitly, or their articulation is not captured through some failure of the research process. These types of gap *do* represent incompleteness and skew the map’s representation of the ethical landscape. This can be counteracted with a bottom–up analysis, where actors have the opportunity to respond to any principle they see fit. The same iterative development should be adopted; as one actor identifies more principles, other actors may wish to make a further response.

Once the participative map has been constructed, a further extension could deploy the methodology as a deliberative tool. This would allow the project aims to extend to proactive engagement with actors and developments. Minimally, this would involve entering discussions with actors about their framing in relation to those of others. This could enhance understanding amongst actors and encourage more robust/sustainable development paths. A more involved approach could open a forum in which actors discuss the technology and its ethical implications together.

To conclude, the methodology described can be used to construct and analyse a map of the ethical landscape of a technology under resource constraints. This can be used to identify features of the ethical landscape and scope for areas where ethical contentions may arise. However, the map has a potential second function, as a pilot study for a participative, deliberative analysis with grander aims.
